# Diffusion and distribution of deuterium in scandium deuteride thin films under irradiation of deuterium ion beam

**DOI:** 10.1038/s41598-017-13780-8

**Published:** 2017-10-17

**Authors:** Tao Wang, Jidong Long, Shiwei Wang, Zhen Yang, Jie Li, Gang Huang, Linwen Zhang, Allen Jian Yang, Xiao Renshaw Wang

**Affiliations:** 1 Institute of Fluid Physics, CAEP, P. O. Box 919-106, Mianyang, 621900 China; 2 School of Nuclear Science and Technology, University of Science and Technology of China, Hefei, 230026 China; 3 School of Sino-Russian, Heilongjiang University, Harbin, 150000 China; 4 Division of Physics and Applied Physics, School of Physical and Mathematical Sciences, Nanyang Technological University, Singapore, 637371 Singapore; 5 School of Electrical and Electronic Engineering, Nanyang Technological University, Singapore, 639798 Singapore

## Abstract

Scandium deuteride (ScD_x_) thin films, as an alternative target for deuterium-deuterium (D-D) reaction, are a very important candidate for detection and diagnostic applications. Albeit with their superior thermal stability, the ignorance of the stability of ScD_x_ under irradiation of deuterium ion beam hinders the realization of their full potential. In this report, we characterize ScD_x_ thin films with scanning electron microscopy (SEM) and X-ray diffraction (XRD), Rutherford backscattering spectroscopy (RBS) and elastic recoil detection analysis (ERDA). We found with increased implantation of deuterium ions, accumulation and diffusion of deuterium are enhanced. Surprisingly, the concentration of deuterium restored to the value before implantation even at room temperature, revealing a self-healing process which is of great importance for the long-term operation of neutron generator.

## Introduction

Detection and diagnostic techniques employing neutron beams are extensively used for quality control in advanced manufacturing, contraband detection, advanced medicine, oil well logging, etc.^1–8^. As an indispensable component of such kind of techniques, neutron generators take advantage of the deuterium-deuterium (D-D) reaction which generates a controllable high-flux neutron beam from a small source. This reaction is commonly triggered by bombarding a metal deuteride target with accelerated deuterium ions^9^. Thus the type and quality of the metal deuteride targets are of great importance to the D-D reaction. In the past two decades, various metal deuteride targets have been studied^10–19^. Nevertheless, they have a variety of shortcomings. For instance, titanium deuteride thin films, which are considered as the most promising target for D-D reaction, suffer from unsatisfactory thermal stability.

Scandium deuteride (ScD_x_), as an alternative target for D-D reaction, has been drawing increasing attention because of its superior thermal stability^17–23^. However, little is known about the content and distribution of deuterium in ScD_x_ thin films when they are subject to irradiation of deuterium ion beams although they are crucial to the stable operation of neutron generators. Herein, we report the characterization of ScD_x_ thin films under irradiance of deuterium ion beams, which reveals the accumulation of deuterium at the ion-implanted region and the subsequent rapid diffusion of deuterium at room temperature. We believe this reinstallation is a previously unknown self-healing process which is crucial to the stable operation of neutron generators based on D-D reaction.

## Experimental Details

### Preparation of ScD_x_ films

The molybdenum substrates were polished, rinsed with a mixed acid (nitric acid and sulfuric acid), washed in deionized water, cleaned in acetone ultrasonically for 30 min, and dried under a nitrogen atmosphere. The ScD_x_ thin films were grown by one-step reactive magnetron sputtering (Magnetron sputtering machine produced by Beijing Technol Science Co. Ltd). High purity argon gas (99.999%) and deuterium gas (99.999%) were used as the sputtering gas and reactive gas, respectively. A metallic Sc target of high purity (99.95%) was utilized to deposit the ScD_x_ films^24–26^. The base pressure was about 10^−5^ Pa. The total pressure of the (Ar + D_2_) gas mixture was set at 0.4 Pa, while the rate of Ar flow and D_2_ flow were 5 SCCM and 15 SCCM respectively. Two-micrometer-thick ScD_x_ thin films were deposited on Mo substrates at 573 K substrate temperature and −250 V DC substrate bias.

### Irradiation experiment

The irradiation experiment was carried out with a multi-voltage accelerator. Deuterium ions produced by the ion source were accelerated with a voltage of 120 kV and implanted into the as-grown ScD_x_ thin films. Experimental parameters can be seen in the Table 1. Sample S1 is the as-grown sample, while samples S2 and S3 are irradiated by deuterium of different doses.

**Table 1:** Parameters of irradiation experiments.

Sample number	Ion implanted	Energy	Ion current density	Dose
S1	/	/	/	/
S2	deuterium	120 keV	10 μA/cm^2^	1*10^17^ atoms/cm^2^
S3	deuterium	120 keV	10 μA/cm^2^	5*10^17^ atoms/cm^2^

### Characterization methods

A Phenom desktop G2 pro scanning electron microscope (SEM) was used to observe the surface morphology of the film surface. The X-ray diffraction (XRD) profiles were recorded using Cu Kα radiation on X’ Pert PRO that operated at 40 kV and 40 mA. The step size, angular range and counting time are 2*θ* = 0.02°, 2*θ* = 20–70° and 1 second, respectively.

Elastic recoil detection analysis (ERDA) was employed to determine deuterium concentrations using the NEC 9SDH-2 × 3 MV pelletron tandem accelerator at Fudan University. The chamber background pressure during ion beam analysis was lower than 1 × 10^−4^ Pa. The deuterium depth profiles in the samples were investigated by ERDA using a 4.5 MeV helium ion beam with a 75° incident angle with respect to the normal direction of the sample surface. A mylar foil with a thickness of 19 μm in front of the detector was used to absorb scattered helium. In addition, the composition of the film was determined by Rutherford Backscattering Spectroscopy (RBS) with 4.5 MeV helium ions beam perpendicular to the surface of the sample. The ERDA spectra were converted into deuterium concentration profiles by Alegria 1.0 code^27^ with an expected systemic error below 7%. The atomic composition of the layer of the sample was given by RBS spectrum unfolding results analyzed with SIMNRA 6.03 code^28^. Based on the RBS and ERDA measurements, we obtained the atomic ratio of D/Sc^29–31^.

## Results and Discussion

**Figure 1:**
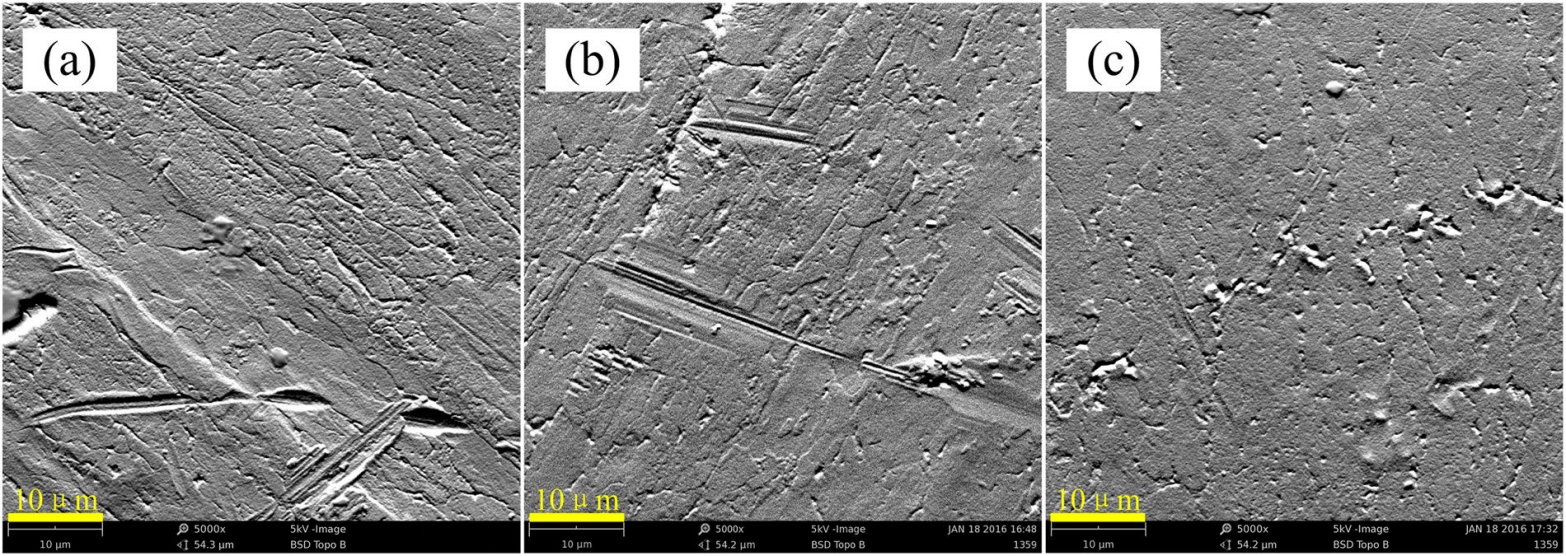
Surface morphologies of the as-grown sample (**a**) and ion-implanted samples (**b**,**c**).

Figure 1 shows SEM images of the as-grown sample and implanted samples, demonstrating the microscopic irregularities on the surface. However, there is no observable morphological difference between the as-grown and irradiated samples, suggesting little mechanical damage due to the ion implantation.

**Figure 2:**
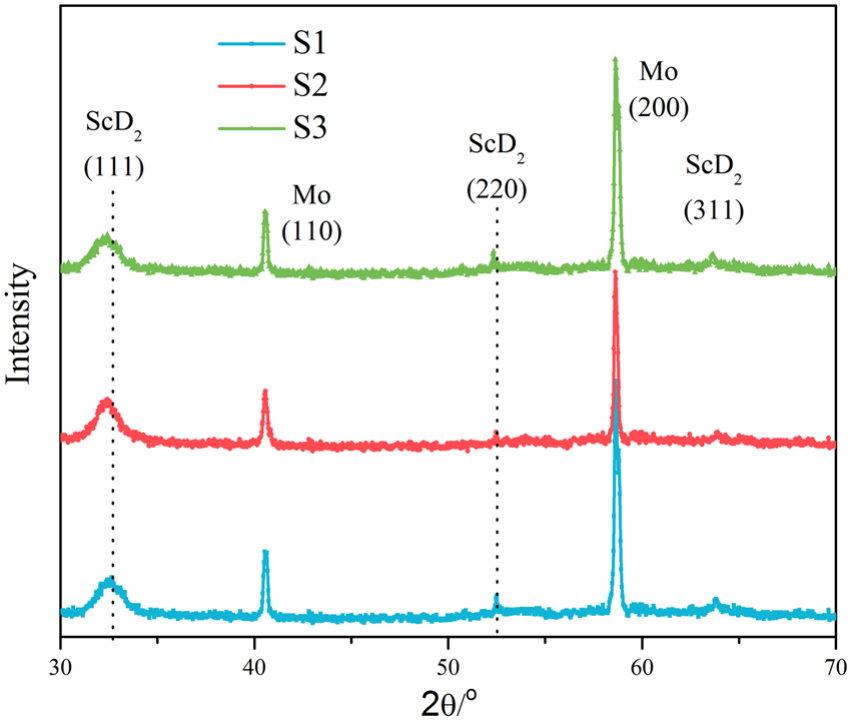
XRD patterns of the scandium deuteride films after irradiation.

Figure 2 shows the XRD patterns of the scandium deuteride thin films with and without deuterium ion irradiation. The structural analysis mainly indicates that the film has ScD_2_ phase and substrate Mo phase. The intensities of ScD_2_ phase peaks remain constant but the positions of these peaks shift to a smaller angle for the irradiated samples. It means that the lattice constants of scandium deuteride films increase after deuterium irradiation. No Sc phase peak is observed, suggesting that deuterium did not desorb from scandium deuteride film because the samples are water-cooled to prevent temperature rise.

**Figure 3:**
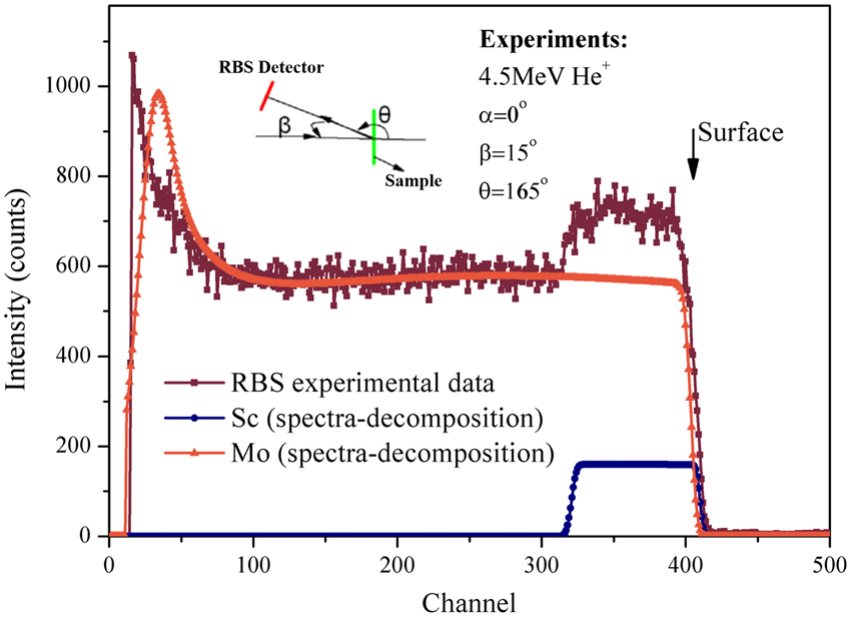
Energy spectrum of He^+^ particles scattered from ScD_x_/Mo film.

A typical energy spectrum of He^+^ particles scattered from ScD_x_/Mo film during irradiation of 4.5 MeV He^+^ with 0° incident angle is presented in the Fig. 3. The horizontal axis corresponds to the energy of the scattered He^+^, namely the depth from the specimen surface to the bulk. The energy spectrum profile reflects the structure of the layered ScD_x_/Mo thin film, and thickness of the film. Apart from the Mo substrate and the top ScD_x_ thin film, no other elements are detected, proving the high purity of the grown ScD_x_ thin film.

**Figure 4:**
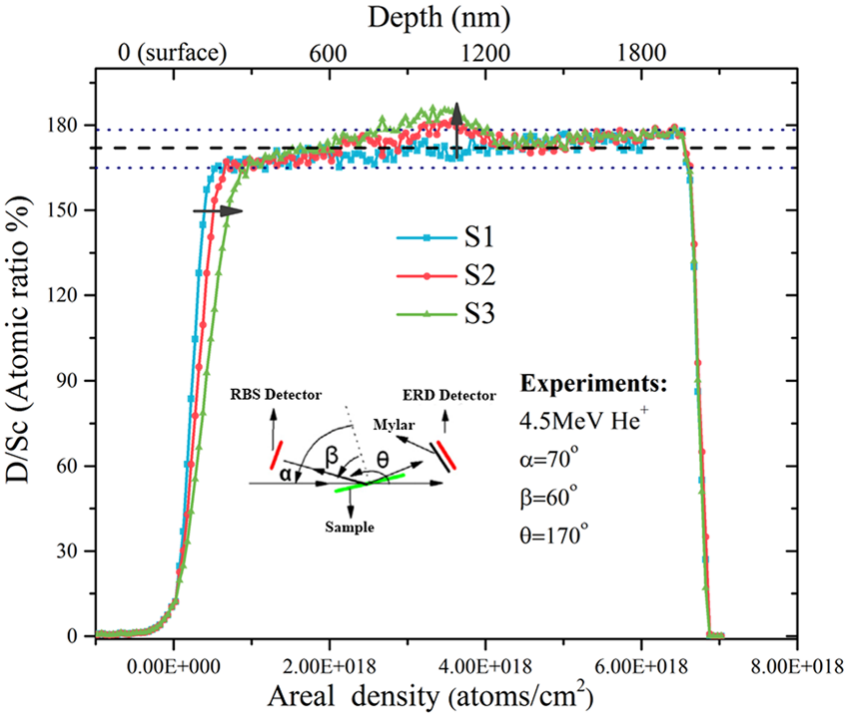
Deuterium concentration of S1, S2 and S3 with different depth in the film.

The deuterium concentrations (atomic ratio of D to Sc) of scandium deuteride films, as shown in Fig. 4, were calculated from the standard analyses of ERD and RBS spectra. The lower horizontal axis corresponds to the areal density of scandium deuteride film, namely the distance from the sample surface to the bulk (about 2 μm), which is indicated by the upper horizontal axis. The vertical axis corresponds to the deuterium concentration in the scandium deuteride film. The average D/Sc atomic ratio is 1.74 ± 0.08.

**Figure 5:**
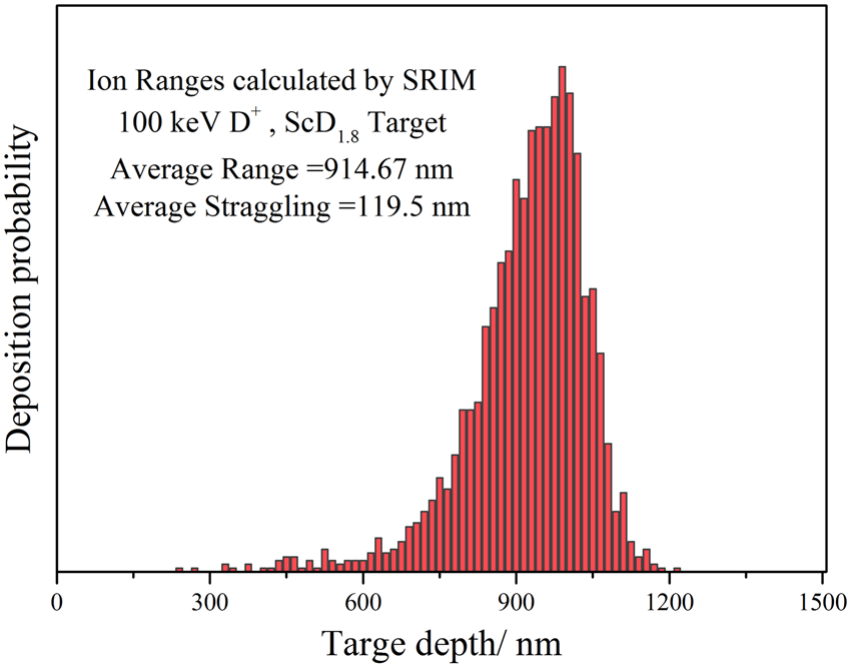
Deuterium deposition peak calculated by SRIM results.

After deuterium ion implantation, an additional peak corresponding to the deuterium content occurs at about 600–1200 nm. This is the deposition area of the incident deuterium ion. In comparison with the Stopping and Range of Ions in Matter (SRIM) results (Fig. 5), the additional peak position of deuterium content is similar to the deposition peak calculated by SRIM. However, the additional peak is slightly broadened due to deuterium diffusion from high concentration area to low concentration area in the film. As seen in the Fig. 4, the broadening is greater at the surface of the film. This phenomenon is probably due to the dependency of diffusion rate on the temperature. Forced water cooling on the back end of the target weakens the diffusion of deuterium into the film. The surface thermal effect of the film is more obvious to promote the diffusion of deuterium to the surface.

With the increase of the deuterium ion implantation, the deuterium accumulation in the deposition region and the deuterium diffusion are more obvious. Surprisingly the deuterium content of the film surface decreases with the increase of the number of implanted ion. We speculate that thermal effects will increase the deuterium equilibrium pressure at the surface, which leads to deuterium desorption of the surface. At the same time, the radiation damage caused by a large number of deuterium ions decreases the migration rate of deuterium in the film.

**Figure 6:**
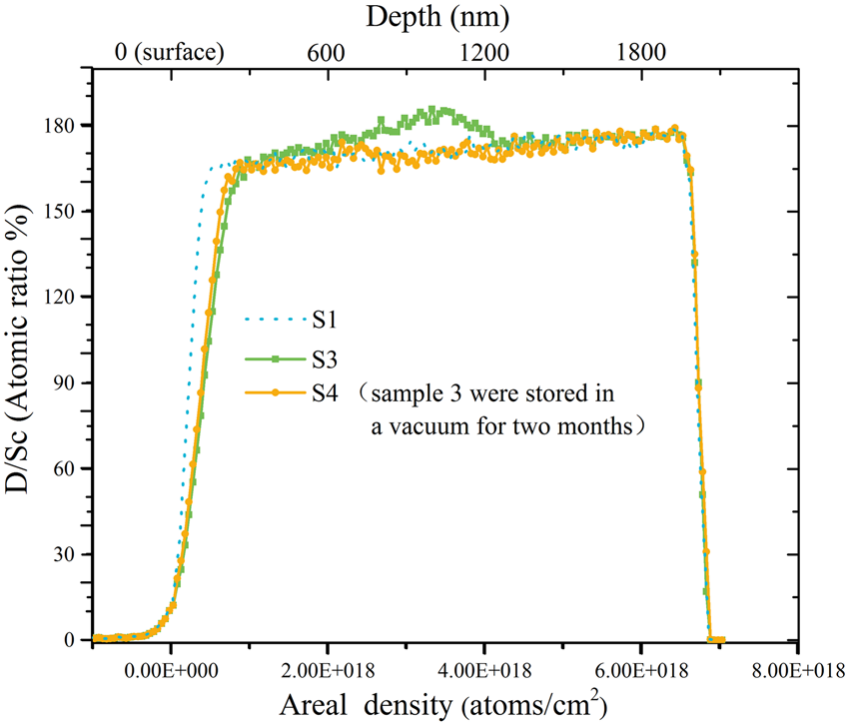
Deuterium concentration of S1, S3 and S4 with different depth in the film.

Sample S3 was stored in a vacuum storage vessel (10^−3^ Pa, 300 K) for two months (called sample S4) and then characterized again. Figure 6 shows the deuterium concentration as a function of depth in the film. Surprisingly, deuterium in the deposition region is fully diffused and the whole film is basically in equilibrium. In contrast to Ar and O, deuterium diffuses rapidly at room temperature. Moreover, the concentration of deuterium is restored to the original value before D-D reaction, implying a self-healing capability. We hypothesize that this is due to the fact that the defects have a stronger pinning effect on the deuterium atom, which is much more important than the diffusion effect based on the chemical potential difference^32–35^. Besides, there is a slight increase in the surface deuterium content. This indicates that the diffusion of deuterium is weakened at the surface area. Compared to sample S1, the deuterium contents of S3 and S4 decrease significantly at the surface of the film.

## Conclusions

We have carried out SEM, XRD, RBS and ERDA studies of the diffusion and distribution of deuterium in ScD_x_/Mo thin films before and after irradiation of deuterium ions. With the increase of the deuterium ion implantation, the deuterium accumulation in the deposition region and the deuterium diffusion is more obvious. It is found that deuterium diffuses rapidly at room temperature and the concentration of deuterium at the implanted region returns to the original state. Such a self-healing effect is of great importance for the long-term stability of neutron generators based on D-D reaction.
